# How physical exercise influences children’s social-emotional competence: an empirical study based on a chain mediation model

**DOI:** 10.1186/s40359-026-04540-3

**Published:** 2026-04-15

**Authors:** Menghua Wang, Qin Zeng, Wenbin Wu, Kelei Guo, Rong Xie

**Affiliations:** 1 Zhaoqing University, Zhaoqing, Guangdong China; 2 Guangzhou Sport University, Guangzhou, China; 3 Guangdong Nanhua Vocational College of Industry And Commerce, Guangzhou, Guangdong China

**Keywords:** Physical exercise, Social-emotional competence, Psychological resilience, Self-efficacy, Children

## Abstract

Social-emotional competence plays a crucial role in children’s development. The present study aimed to examine the effect of physical exercise on children’s social-emotional competence and to test the mediating roles of psychological resilience and self-efficacy in this relationship. Data were collected from 331 children (10.07 ± 1.14 years) in Guangdong Province, China, through a questionnaire survey assessing physical exercise, psychological resilience, self-efficacy, and social-emotional competence. The results showed that physical exercise was a significant positive predictor of children’s social-emotional competence. Both psychological resilience and self-efficacy exerted significant mediating effects between physical exercise and social-emotional competence. Specifically, three mediation pathways were identified: (1) the independent mediating effect of psychological resilience between physical exercise and social-emotional competence; (2) the independent mediating effect of self-efficacy between physical exercise and social-emotional competence; and (3) the chain mediating effect of psychological resilience and self-efficacy in the relationship between physical exercise and social-emotional competence. These findings elucidate the underlying mechanisms through which physical exercise influences children’s social-emotional competence and provide theoretical and practical evidence for promoting social-emotional competence in children.

## Introduction

Social-emotional competence refers to a comprehensive set of abilities that enable individuals to recognize and manage their own emotions, establish and maintain positive social relationships, and make responsible decisions [1]. It constitutes a critical foundation for early childhood development as well as for the acquisition of more complex skills later in life, and it exerts a profound influence on individuals’ functioning in adulthood [2]. Empirical evidence indicates that children with well-developed social-emotional competence are more likely to form harmonious interpersonal relationships, maintain better mental health, and achieve higher academic performance compared with their peers [3]. In contrast, deficits in social-emotional competence are associated with increased negative emotional experiences, such as anxiety and insecurity, as well as a significantly elevated risk of problem behaviors [4]. However, the development of social-emotional competence among children worldwide is currently facing a widespread and pressing challenge. Data from the United States show that 55%–71% of surveyed students exhibit substantial shortcomings in key dimensions of social-emotional competence, including empathy, decision-making ability, and interpersonal skills, while 7%–24% of young children demonstrate delayed development in social-emotional competence [5]. In China, approximately 13.3% of children display problem behaviors, which are significantly associated with delayed development of social-emotional competence [6].

Against this backdrop, social-emotional competence, as a core competency for children and adolescents in the 21st century [7], has attracted increasing attention from the global academic community as well as from international organizations such as the United Nations Educational, Scientific and Cultural Organization [8], OECD [9], and the World Bank [10]; Extensive research and practice have been conducted on its measurement, cultivation, and underlying mechanisms. Nevertheless, identifying effective and scalable approaches to enhance children’s social-emotional competence—particularly practical, everyday intervention strategies and their internal mechanisms—remains a central challenge in the field. Physical exercise, as a process rich in emotional experience, moral development, and character building, holds irreplaceable value in cultivating children’s social-emotional competence [11, 12]. Although existing research has revealed the association between physical exercise and social-emotional competence, the underlying mechanisms still need further clarification. A deeper exploration of these mechanisms is crucial for understanding the development of children’s social-emotional competence and optimizing related intervention strategies. Positive psychology points out that positive psychological qualities are important factors in promoting children’s development [13]. As important positive psychological qualities related to physical exercise, psychological resilience and self-efficacy reflect an individual’s ability to adapt when facing challenges and their cognitive assessment of their own capabilities. Moreover, this adaptability often influences an individual’s cognitive assessment [14], playing distinct but complementary roles in the process of social-emotional development [15, 16]. Therefore, this study introduces psychological resilience and self-efficacy as mediator variables, constructing a serial mediation model to systematically reveal the potential pathways between physical exercise and children’s social-emotional competence from a positive psychology perspective, with the aim of providing theoretical and practical guidance for promoting the development of contemporary children’s social-emotional competence.

### The relationship between physical exercise and children’s social-emotional competence

Mantz conceptualized children’s social-emotional competence as comprising four dimensions: social awareness, self-management, responsible decision-making, and interpersonal skills [17]. In existing studies, physical exercise can serve as an effective approach to promoting the coordinated development of these abilities. First, as an important component of social awareness, empathy can be significantly enhanced through physical exercise, thereby facilitating the positive development of children’s social awareness [18]. Second, during physical exercise, children are required to continuously cope with negative emotional challenges caused by factors such as fatigue and failure. This process helps to train and improve their emotion regulation ability, thus providing a psychological basis for the further development of self-management [19]. With respect to decision-making ability, the study by Bulger provides an example: children were able to autonomously adjust their movement strategies according to target distance during throwing practice, reflecting the role of physical exercise in promoting decision-making and problem-solving abilities [20, 21]. In addition, participation in physical exercise helps to enhance cohesion within children’s groups and to build more positive interpersonal interaction networks [22]. By improving children’s self-control and empathy, physical exercise can effectively reduce interpersonal conflicts and thereby significantly promote the development of interpersonal skills [23, 24].

Based on this, the present study proposes Hypothesis 1: Physical exercise positively affects children’s social-emotional competence.

### The mediating effect of psychological resilience

One of the mediating mechanisms examined in this study is the mediating role of psychological resilience. Psychological resilience refers to an individual’s ability to adapt to change and stress in a healthy and adaptive manner [25], It comprises four dimensions: control (the individual’s internal tendency to regulate their own behavior and influence the environment), commitment (the tendency to actively engage in a group rather than remain isolated from it), challenge (the belief that events are changeable and that situations should be viewed as opportunities rather than threats), and confidence (the individual’s firm belief in their own ability to achieve success) [26]. Marshall et al. found, through a one-month exercise intervention, that participants showed significant increases in the confidence and commitment dimensions. Moreover, individuals who demonstrated greater improvements in motor performance during physical exercise exhibited more pronounced enhancements in psychological resilience [27], providing evidence that psychological resilience can be acquired and strengthened through regular physical exercise [28, 29]. In addition, a significant association exists between psychological resilience and children’s social-emotional competence, such that higher levels of psychological resilience are associated with higher overall levels of social-emotional competence [30]. Previous studies have shown that psychological resilience, as an important predictor of emotion regulation, plays a critical role in alleviating negative emotional states in children, including tension, anxiety, and depression [31], thereby positively influencing children’s self-management ability. Furthermore, the four-dimensional model of psychological resilience (control, commitment, challenge, and confidence) corresponds to task performance (self-control and perseverance), interpersonal skills (sociability), and emotion regulation (optimism) in the OECD social-emotional competence framework [32]. Individuals with higher psychological resilience tend to score higher on task performance dimensions [33], providing empirical support for the association between the two constructs.

Based on this, Hypothesis 2 is proposed: Psychological resilience mediates the effect of physical exercise on children’s social-emotional competence.

### The mediating effect of self-efficacy

Another mediating mechanism examined in this study is the mediating role of self-efficacy. Self-efficacy, first proposed by Bandura, refers to individuals’ confidence or belief in their ability to achieve specific behavioral goals within a given domain [34]. Research has shown that physical exercise is an important pathway for enhancing self-efficacy. On the one hand, physical exercise positively predicts self-efficacy by strengthening individuals’ physical self-identity [35, 36]. On the other hand, according to self-efficacy theory, mastery experiences are the core source of self-efficacy [37]. The process through which children acquire motor skills and knowledge during physical exercise constitutes an important form of mastery experience, thereby effectively enhancing their self-efficacy [38]. Self-efficacy is not only a core variable within the individual motivational system but is also regarded as a key factor influencing the development of children’s social-emotional competence [39]. Children with high self-efficacy demonstrate better communication skills, problem-solving abilities, and confidence in interpersonal interactions, which significantly promote the establishment and maintenance of positive interpersonal relationships [40]. At the mechanistic level, self-efficacy, as an important psychological resource for coping with social situations, can significantly enhance children’s adaptability and regulatory capacity in social interactions [41]. When facing stress or challenges, individuals with higher levels of self-efficacy are better able to manage their emotions and respond proactively, thereby exhibiting higher levels of social-emotional competence [42, 43].

Based on this, Hypothesis 3 is proposed: Self-efficacy mediates the effect of physical exercise on children’s social-emotional competence.

### The chain mediating effects of psychological resilience and self-Efficacy

Another mediating mechanism examined in this study is the chain mediating effect of psychological resilience and self-efficacy. Previous research has shown that psychological resilience is one of the key factors enhancing self-efficacy. As an internal resource for coping with stress and challenges, psychological resilience helps individuals maintain a positive psychological state, thereby strengthening their confidence in their own abilities [44]. When encountering adversity or challenging situations, children with higher levels of psychological resilience tend to exhibit greater confidence and perseverance. Such positive coping facilitates the acquisition of actual mastery experiences [42, 45]. and these success experiences derived from personal effort represent a crucial pathway for activating and enhancing self-efficacy [46]. An individual’s self-efficacy is dynamically influenced by multiple factors, including ability appraisal, perceived task difficulty, effort investment, and past experiences [47]. As a protective mechanism, psychological resilience is typically associated with firm self-beliefs. Individuals with higher psychological resilience are more likely to make positive evaluations of their own abilities, task difficulty, and effort expenditure, thereby further enhancing their self-efficacy [48].

Based on this, Hypothesis 4 is proposed: Psychological resilience and self-efficacy may jointly play a chain mediating role in the relationship between physical exercise and children’s social-emotional competence.

**Fig. 1:**
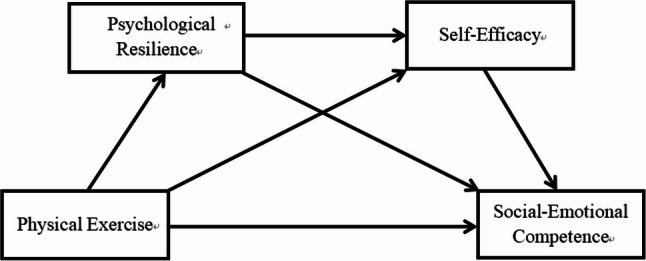
Conceptual Framework

In summary, to examine the mechanisms underlying the relationship between physical exercise and children’s social-emotional competence, the present study proposes a chain mediation model (see Fig. 1) and aims to test the following hypotheses: (1) physical exercise significantly and positively predicts children’s social-emotional competence; (2) psychological resilience and self-efficacy independently mediate the relationship between physical exercise and children’s social-emotional competence; and (3) psychological resilience and self-efficacy jointly exert a chain mediating effect in the relationship between physical exercise and children’s social-emotional competence.

## Research methods

### Participants

This study employed a convenience sampling method, selecting three primary schools from first, second, and third-tier cities in Guangdong Province, China, as the sample source, covering students from different grade levels. The students from the selected schools exhibit diversity in basic demographic variables such as gender and grade level, ensuring a certain degree of diversity in the sample. Data collection was conducted between March 1 and March 31, 2025. The inclusion criteria were as follows: (1) enrollment as a student in Grades 3 to 6; (2) good physical health with no motor impairments; and (3) absence of cognitive impairments, with the ability to accurately understand the questionnaire items and instructions. Individuals who did not meet these criteria were excluded from the study. A total of 360 children who met the inclusion criteria were each administered one integrated questionnaire consisting of four scales. After data screening (e.g., missing responses and duplicate submissions), 29 invalid questionnaires were excluded, resulting in a final valid sample of 331 questionnaires, with an effective response rate of 91.94%. The average age of the subjects was 10.13 ± 1.405 years. Among the participants, 179 were boys (54%) and 152 were girls (45%). The sample included 70 third-grade students (21.1%), 84 fourth-grade students (25.3%), 86 fifth-grade students (25.9%), and 91 sixth-grade students (27.4%). The questionnaires were administered in paper-and-pencil format and distributed by the research team. Data collection took place during self-study periods in a classroom setting. Prior to questionnaire administration, the researchers provided participants with a detailed explanation of the study purpose, clear instructions for completion, and addressed any questions or concerns raised by the participants. Each participant was required to complete an integrated questionnaire comprising four scales: the Physical Activity Rating Scale, the Social-Emotional Competence Scale, the Psychological Resilience Scale, and the Self-Efficacy Scale. The questionnaire consisted of a total of 52 items, with an estimated completion time of approximately 8 min.

Before data collection, all researchers received centralized training to ensure standardized procedures. During the field investigation, researchers supervised the questionnaire completion process, informed participants of relevant study information, and obtained their informed consent along with written assent. As the participants were minors, written consent was also obtained from their legal guardians. In addition, the study protocol was reviewed and approved by the Human Research Ethics Committee of Guangzhou Sport University (approval number: 2024LCLL-72). The questionnaire instructions emphasized anonymity, assured participants that there were no right or wrong answers, clarified that the data would be used solely for scientific research purposes, and specified the expected completion time.

### Measurement tools

#### Physical activity rating scale

The study employed the Physical Activity Rating Scale, initially developed by Hashimoto et al. (1990) and subsequently adapted into Chinese by Liang Deqing et al. [49]. This instrument assesses physical activity across three dimensions: intensity, duration, and frequency. Each dimension comprises five graded levels. The overall physical activity level score is derived from the formula: Activity Level Score = Intensity × (Duration − 1) × Frequency. For example, if exercise duration = 4 (score of 3), exercise intensity = 4 (score of 4), and exercise frequency = 5 (score of 5), then the physical exercise level is calculated as 3 × 4 × 5 = 60. Previous studies have demonstrated that this scale is suitable for use with child populations [50, 51]. In the present study, the scale showed acceptable internal consistency, with a Cronbach’s α coefficient of 0.705.

#### Social emotional competence scale

Social emotional competence was measured using the Chinese version of the Delaware Social-Emotional Competence Scale [52]. The scale consists of 12 items and comprises four dimensions: self-management, interpersonal relationships, social awareness, and responsible decision-making, with three items in each dimension. Responses are rated on a 4-point Likert scale, ranging from 1 (“not at all like me”) to 4 (“very much like me”), with intermediate options of 2 (“not very much like me”) and 3 (“somewhat like me”). The items primarily reflect the developmental level of children’s social-emotional competence, including self-management ability, interpersonal skills, social awareness, and the ability to make responsible decisions. Previous studies have demonstrated that this scale is suitable for use with child populations [3, 53]. In the present study, the scale demonstrated acceptable internal consistency, with a Cronbach’s α coefficient of 0.724.

#### Child resilience scale

Psychological resilience was assessed using the Adolescent Psychological Resilience Scale [54], developed by Hu Yueqin and Gan Yiqun. The scale comprises five dimensions: positive cognition, goal focus, family support, interpersonal assistance, and emotion control, with a total of 27 items, of which 12 are reverse-scored. Responses are rated on a 5-point Likert scale, with higher scores indicating higher levels of psychological resilience. Previous studies have demonstrated that this scale is suitable for use with child populations [55, 56]. In the present study, the scale demonstrated acceptable internal consistency, with a Cronbach’s α coefficient of 0.701.

#### Self-efficacy scale

Self-efficacy was evaluated using the Chinese version of the General Self-Efficacy Scale (GSES), revised by Wang Caikang et al. (2001) [57]. The scale does not distinguish between dimensions and consists of 10 items. Responses are rated on a 4-point Likert scale, ranging from 1 (“completely inconsistent”) to 4 (“completely consistent”), with higher scores indicating higher levels of self-efficacy. Previous studies have demonstrated that this scale is suitable for use with child populations [58, 59]. In the present study, the scale showed good internal consistency, with a Cronbach’s α coefficient of 0.851.

### Data processing

Data analysis was performed using SPSS 26.0 for Pearson correlation analysis and the PROCESS macro for mediation effect testing. Confirmatory factor analysis (CFA) of the questionnaires was conducted using Amos 29.0.

## Research results and analysis

### Common method bias test

As data in the present study were collected using self-report measures, the potential for common method variance (CMV) could not be ruled out. Therefore, necessary procedural controls were implemented during data collection, such as informing participants in the questionnaire instructions that the survey was anonymous and that the data would be used solely for scientific research. In addition, some questionnaire items were reverse-scored. To assess common method bias, Harman’s single-factor test was conducted. The results of the unrotated exploratory factor analysis (EFA) indicated that 15 factors with eigenvalues greater than 1 were extracted, and the first factor accounted for 17.087% of the total variance, which is below the critical threshold of 40%. Furthermore, this study used AOMS software to conduct a single-factor confirmatory factor analysis to test common method bias (CMB). In this analysis, all measurement items were forced to load onto the same latent factor, and the model was treated as a constrained model to evaluate the presence of a single method factor. The results showed that the fit of the single-factor model did not meet good fit criteria (see Table 1), indicating that a single factor could not explain the covariance relationships between the observed variables. This result suggests that the data were not dominated by a single method factor, thus supporting the conclusion that there is no severe common method bias in this study.

**Table 1:** Model Fit Indices

Fit Index	Model Value	Criterion
x^2^/df	3.034	< 3.000, good fit
CFI	0.457	> 0.900, good fit
TLI	0.435	> 0.900, good fit
IFI	0.463	> 0.900, good fit
RMSEA	0.089	< 0.080, good fit
SRMR	0.092	< 0.080, good fit

### Descriptive statistics and correlation analysis of variables

As shown in Table 2, the correlation coefficients among physical exercise, psychological resilience, self-efficacy, and social-emotional competence were all positive and statistically significant. Among these variables, physical exercise showed the strongest correlation with social-emotional competence (*r* = 0.426). The correlation between self-efficacy and social-emotional competence was slightly lower (*r* = 0.422). Psychological resilience was moderately and positively correlated with self-efficacy (*r* = 0.271) and social-emotional competence (*r* = 0.292), while physical exercise was also moderately and positively correlated with psychological resilience (*r* = 0.247) and self-efficacy (*r* = 0.297).

**Table 2:** Means, Standard Deviations, and Correlation Coefficients for All Variable

Variable	M	SD	1	2	3	4
PE	54.91	30.171	1			
PR	84.28	11.808	0.247**	1		
SE	34.94	7.230	0.297**	0.271**	1	
SEC	36.08	4.990	0.426**	0.292**	0.422**	1

N = 331

*PE *Physical Exercise*, PR * Psychological Resilience*, SE *Self-Efficacy*, SEC * Social-Emotional Competence

* p < 0.05, ** p < 0.01

These results provide preliminary evidence suggesting that psychological resilience and self-efficacy may play mediating roles in the relationship between physical exercise and children’s social-emotional competence.

### Mediating effects of resilience and self-efficacy

**Fig. 2:**
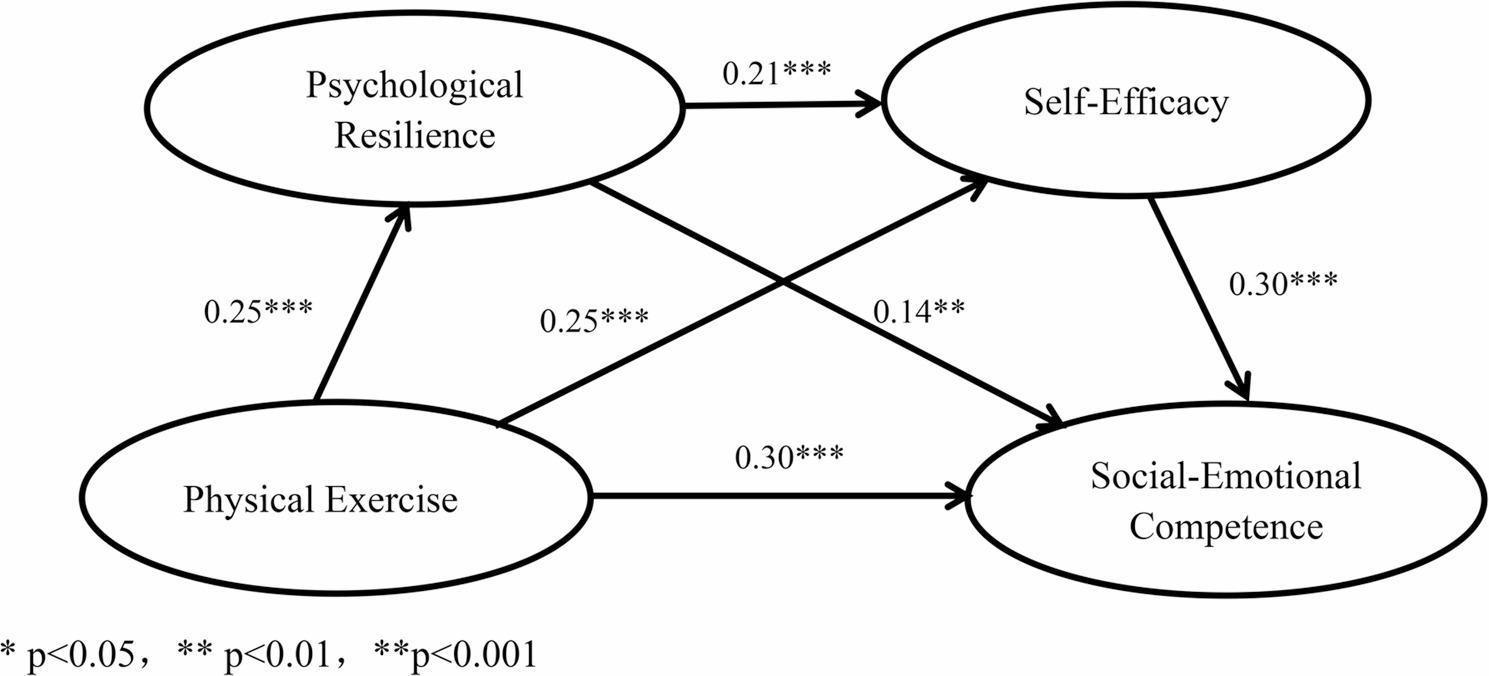
Chain Mediation Model of Physical Exercise and Social-Emotional Competence

The present study employed standardized data and utilized the PROCESS macro (Model 6) developed by Hayes in SPSS to construct and test the serial mediation model. The path coefficients are presented in Fig. 2. As shown in Table 3, the overall regression model was significant (R² = 0.18, F = 72.82, *p* < 0.001). The mediation effects were tested using 5,000 bootstrap samples, and bias-corrected (BC) 95% confidence intervals were generated. The results indicated that the total effect of physical exercise on social-emotional competence was 0.43. Path analysis revealed that physical exercise positively predicted psychological resilience (β = 0.25, t = 4.63, *p* < 0.001), self-efficacy (β = 0.25, t = 4.60, *p* < 0.01), and social-emotional competence (β = 0.30, t = 6.15, *p* < 0.001). Psychological resilience positively predicted both self-efficacy (β = 0.21, t = 3.96, *p* < 0.001) and social-emotional competence (β = 0.14, t = 2.79, *p* < 0.01), while self-efficacy positively predicted social-emotional competence (β = 0.30, t = 5.92, *p* < 0.001). All regression equations reached statistical significance. Furthermore, the explained variance (R²) for each regression model was as follows: physical exercise explained 6% of the variance in psychological resilience (R² = 0.06, F = 21.45, *p* < 0.001); physical exercise and psychological resilience together explained 13% of the variance in self-efficacy (R² = 0.13, F = 24.44, *p* < 0.001); and physical exercise, psychological resilience, and self-efficacy jointly explained 29.4% of the variance in social-emotional competence (R² = 0.294, F = 45.42, *p* < 0.001).

**Table 3:** Analysis of Regression Relationships Among Variables

Variables	Psychological Resilience		Self-Efficacy		Social-Emotional Competence		Total Effect	
	β	t	β	t	β	t	β	t
Physical Exercise	0.25	4.63	0.25	4.60	0.30	6.15	0.43	8.53
Psychological Resilience			0.21	3.96	0.14	2.79		
Self-Efficacy					0.30	5.92		
R²	0.03		0.13		0.29		0.18	
F	21.45***		24.44***		45.42***		72.82***	

* *p* < 0.05, ** *p* < 0.01, **p < 0.001

### Analysis of mediation effects

The results in Table 4 indicated that the total indirect effect was 0.121 (95% CI: 0.067, 0.189), suggesting that the serial mediation model involving psychological resilience and self-efficacy was supported. After including psychological resilience and self-efficacy, the direct effect was 0.304 (95% CI: 0.207, 0.402), indicating that psychological resilience and self-efficacy partially mediated the relationship between physical exercise and social-emotional competence. The total indirect effect (0.121) accounted for 28.52% of the total effect (0.426). Specifically, the mediation effect consisted of three pathways: (1) indirect path 1 (physical exercise → psychological resilience → social-emotional competence), (2) indirect path 2 (physical exercise → self-efficacy → social-emotional competence), and (3) indirect path 3 (physical exercise → psychological resilience → self-efficacy → social-emotional competence). The effect sizes of these three paths were 0.034, 0.072, and 0.015, accounting for 7.96%, 16.94%, and 3.62% of the total effect, respectively. The bootstrap 95% confidence intervals for all three indirect paths did not include zero, indicating that all mediation effects were statistically significant. These findings support Hypotheses 2, 3, and 4.

**Table 4:** Mediation Effect Analysis of Physical Activity and Social Emotional Competence

Item	Effect	Boot SE	Bootstrap95% CI		Relative Mediating Effect
			LLCI	ULCI	
Total Effect	0.426	0.050	0.328	0.524	100%
Direct Effect	0.304	0. 050	0.207	0.402	71.48%
Indirect Effect 1	0.034	0.020	0.001	0.079	7.96%
Indirect Effect 2	0.072	0.025	0.030	0.129	16.94%
Indirect Effect 3	0.015	0.008	0.004	0.033	3.62%
Total Indirect Effect	0.121	0.028	0.067	0.189	28.52%

Indirect Effect 1: Physical Exercise → Psychological Resilience → Social-Emotional Competence; Indirect Effect 2: Physical Exercise → Self-Efficacy → Social-Emotional Competence; Indirect Effect 3: Physical Exercise → Psychological Resilience → Self-Efficacy → Social-Emotional Competence

## Discussion

### The impact of physical exercise on children’s social-emotional competence

The findings indicate that physical exercise has a significant direct effect on children’s social-emotional competence, thereby supporting Hypothesis 1. Physical exercise contributes to the enhancement of children’s social-emotional competence through four interrelated pathways: emotion regulation, cognitive decision-making, interpersonal interaction, and social awareness. First, physical exercise provides rich emotional experiences that facilitate the development of children’s emotion regulation abilities. Neuroimaging research has shown that long-term, systematic physical exercise promotes increased cortical thickness in the rostral anterior cingulate cortex of both cerebral hemispheres, thereby optimizing emotion regulation strategies and effectively alleviating negative emotions such as anxiety and tension [60, 61]. Second, physical activity enhances children’s brain structure and cognitive executive functioning. Zimmermann reported that physical exercise not only improves children’s motor decision-making abilities in sports-related contexts but also transfers to everyday life, enhancing the quality of information processing and responsible decision-making [62]. In addition, group games and competitive activities in physical exercise provide children with abundant opportunities for verbal and nonverbal interaction, promoting the development of interpersonal communication and cooperation skills, facilitating the establishment of positive interpersonal relationships, and enhancing social adaptability [63]. More importantly, physical exercise significantly promotes children’s social awareness, defined as the ability to understand and empathize with others’ perspectives. Systematic reviews have shown that sports-based interventions significantly enhance children’s prosocial behaviors, including cooperation, empathy, and altruism, with particularly pronounced effects observed in team-based activities [64]. For example, cooperative sports games and role-rotation settings enhance children’s sensitivity to others’ intentions and emotions, thereby fostering the development of empathy [65].

In summary, physical exercise not only strengthens children’s self-regulation and cognitive decision-making abilities but also promotes the development of social awareness through enriched interactive contexts, ultimately enhancing overall social-emotional competence. These findings provide multi-level empirical support for physical exercise as an effective pathway for promoting the comprehensive development of children’s social-emotional competence.

### The independent mediating effect of psychological resilience

The results indicate that psychological resilience plays a significant mediating role in the relationship between physical exercise and children’s social-emotional competence, thereby supporting Hypothesis 2. This finding suggests that physical exercise not only directly enhances children’s social-emotional competence but also exerts an indirect effect by improving psychological resilience. Specifically, physical exercise is significantly and positively associated with psychological resilience, and psychological resilience is also significantly and positively related to social-emotional competence, which is consistent with previous research findings [66, 67]. As a protective psychological resource that helps individuals cope with external stressors and internal conflicts, psychological resilience is closely related to health-promoting behaviors such as physical exercise [68]. Regular and well-structured physical activity contributes to enhanced functional connectivity between key brain regions, such as the prefrontal cortex and the limbic system [69], thereby improving children’s emotion regulation and behavioral control when facing stress or failure. This process provides strong support for the “self-management” dimension of social-emotional competence. In addition, Maddi noted that psychological resilience is closely associated with hardiness personality traits [70], and physical exercise represents an important pathway for fostering the development of such traits [71]. Repeated exposure to failure, challenge, and physical fatigue in sports contexts encourages children to gradually develop core dimensions of resilience, including control, commitment, challenge, and confidence. This development helps children demonstrate more effective coping strategies when confronted with interpersonal conflict or emotional fluctuation [72], leading to more positive outcomes across social-emotional domains such as emotion regulation, interpersonal skills, and responsible decision-making. Previous studies have also shown that individuals with higher psychological resilience are more likely to maintain an optimistic attitude and engage in positive communication during peer interactions, and to exhibit greater tolerance and empathy when facing divergent opinions or group pressure [73], These characteristics provide a solid psychological foundation for the development of the social awareness dimension of social-emotional competence.

Therefore, by activating children’s internal psychological resilience resources, physical exercise facilitates coordinated development in emotion management and social interaction, ultimately promoting social-emotional competence in a comprehensive manner.

### The independent mediating effect of self-efficacy

The results indicate that self-efficacy plays a significant mediating role in the relationship between physical exercise and social-emotional competence, thereby supporting Hypothesis 3. On the one hand, physical exercise is significantly and positively associated with self-efficacy; on the other hand, self-efficacy is also positively related to social-emotional competence, which is consistent with previous findings [42]. First, regular participation in physical exercise not only significantly enhances children’s self-efficacy related to physical abilities but also exerts a positive influence on general self-efficacy in behaviors such as self-protection and coping with risky situations [74]. In particular, when children demonstrate motor abilities superior to those of their peers during physical exercise, these abilities serve as mastery experiences that promote the development of self-efficacy [75]. At the same time, the sense of pride derived from daily social interactions and activities further strengthens psychological motivation. Driven by this intrinsic motivation, children are more likely to engage actively in physical exercise, exhibiting greater goal persistence and autonomy, which contributes to the improvement of the “self-management” dimension of social-emotional competence. Second, self-efficacy provides essential social and emotional support during children’s development. According to self-efficacy theory, individuals are prone to experience negative emotions such as anxiety, tension, and fear when confronted with difficulties, whereas higher self-efficacy effectively inhibits these negative emotional responses and facilitates more adaptive coping capacities [76]. This process directly contributes to the development of responsible decision-making. Third, high levels of self-efficacy offer children a positive sense of self-identity, helping them regulate conflicts and stress in social behavior, enhancing confidence in interpersonal interactions, and enabling greater initiative and adaptability in interactions with others, thereby promoting the development of interpersonal skills [77]. In addition, children with higher self-efficacy are more likely to develop positive attributional styles, demonstrate greater understanding of others’ emotions and perspectives, and exhibit stronger empathy and social responsibility within group contexts [65]. Such children not only show better emotion regulation but are also more attentive to others’ needs and responses, allowing them to integrate more effectively into social situations.

Therefore, children can enhance their self-efficacy through physical exercise and, in turn, exhibit higher levels of social-emotional competence.

### The serial mediating effect of psychological resilience and self-efficacy

The present study found that physical exercise positively influences children’s social-emotional competence through the chain mediating effects of psychological resilience and self-efficacy, thereby supporting Hypothesis 4. This finding further elucidates the pathway through which physical exercise enhances children’s social-emotional competence by strengthening internal psychological resources. Psychological resilience, as an adaptive capacity developed by individuals in the face of adversity, is significantly and positively associated with self-efficacy [78]. Research has shown that when individuals encounter challenges, psychological resilience helps them identify solutions under pressure and strengthen self-beliefs through repeated successful experiences [79]. Navickienė further indicated that a virtuous cycle exists between psychological resilience and self-efficacy. Psychological resilience cultivated through overcoming challenges and difficulties can effectively enhance self-efficacy; the more frequently individuals overcome adversity, the stronger their psychological resilience becomes, and each successful breakthrough reinforces the belief of “I can do it,” thereby leading to higher levels of self-efficacy [80].

Therefore, the chain mediating mechanism of psychological resilience and self-efficacy clearly reveals the pathway through which physical exercise exerts a deeper influence on social-emotional competence. Physical exercise not only directly improves children’s social-emotional competence but also gradually enhances psychological resilience, which in turn strengthens self-efficacy, ultimately promoting the overall development of children’s social-emotional competence.

### Limitations

This study has several limitations. First, the research design was cross-sectional, which may limit the strength of causal inference. Future research could employ a longitudinal design with multiple time points to explore the dynamic changes and temporal relationships among the variables, thereby providing a more robust basis for causal inferences regarding the mediation pathways proposed in this study. Second, this study examined only two mediating variables—psychological resilience and self-efficacy—in the relationship between physical exercise and children’s social-emotional competence. Whether other potential mediators exist remains to be verified in future research. Third, data were collected using self-report measures, which may involve a certain degree of subjective bias. Future studies could combine self-reports with ratings from others to enhance the objectivity and reliability of the data. Fourth, this study selected children from Guangdong Province, China, as the participants. Due to the influence of local educational and socio-cultural contexts, the applicability of the findings to other regions or backgrounds may be limited. Future research should involve samples from multiple countries and different contexts.

## Conclusions

Based on a cross-sectional study of children in Guangdong Province, China, this study constructed a chain mediation model to examine the relationship between physical exercise and children’s social-emotional competence. The findings provide theoretical support and practical evidence for the cultivation of children’s social-emotional competence. The main conclusions are as follows: (1) physical exercise positively predicts children’s social-emotional competence; and (2) psychological resilience and self-efficacy play a chain mediating role in the relationship between physical exercise and children’s social-emotional competence.

## Data Availability

Due to ethical and privacy considerations, the datasets generated and/or analyzed during the current study are not publicly available but are available from the corresponding author (Rong Xie, 36347565@qq.com) upon reasonable request.

## References

[1] Pu R, Cui X, Qian J. How Family-School Cooperation Affects the Development of the Socio-EmotionalCompetence of Rural Children Left at Home by Their Parents Employed in Cities. Educational Res. 2024;45(06):101–14.

[2] Li L, Hu X, Huang L. The Impact of Family Multidimensional Poverty on Children’s Social-Emotional Competence: An Empirical Study of the Five Western Districts and Counties. J Res Educ Ethnic Minorities. 2024;35(02):85–96. 10.15946/j.cnki.1001-7178.20240506.003.

[3] Wang Y, Xin T, Yang Z, Qin K. The Influence of Social and Emotional Competence Education onChildren in Western China: Empirical Research Based on PropensityValue Analysis. J Chin Soc Educ. 2021;(11):26–31.

[4] Chen Q. Differential Representation of Children’s Social Emotional Ability andCoping Strategies. J Chin Soc Educ. 2021;(11):32–8.

[5] Durlak JA, Weissberg RP, Dymnicki AB, Taylor RD, Schellinger KB. The impact of enhancing students’ social and emotional learning: A meta-analysis of school‐based universal interventions. Child Dev. 2011;82(1):405–32. 21291449 10.1111/j.1467-8624.2010.01564.x

[6] J B-GM SCA, Ornstein DN. Assessment of young children’s social-emotional development and psychopathology: recent advances and recommendations for practice. J Child Psychol Psychiatry Allied Discip. 2004;45(1):109–34. 10.1046/j.0021-9630.2003.00316.x 14959805

[7] Qu L, Chen M. The Perspective Review and Development Orientation of DomesticResearch on the Social and Emotional Skills of Primary and SecondarySchool Students. J Soochow University(Educational Sci Edition). 2023;11(04):42–52. 10.19563/j.cnki.sdjk.2023.04.004.

[8] UNESCO. Creating an enabling learning environment: A comprehensive toolkit on social and emotional learning for preschool educators in developing children’s social and emotional skills n.d. Available from: https://www.unesco.org/en/early-childhood-education/creating-enabling-learning-environment-comprehensive-toolkit-social-and-emotional-learning-preschool

[9] OECD. Social and emotional skills 2024. Available from: https://www.oecd.org/fr/themes/competences-sociales-et-emotionnelles.html

[10] Rogers H. Social and emotional skills: What education leaders need to know. Presentation at the Education Innovations for 21st Century Skills, Bishkek.: World Bank; 2015, June. Available from: https://www.worldbank.org/content/dam/Worldbank/Event/ECA/central-asia/1%20Social%20&%20emotional%20skills__Halsey%20presentation%20for%20Bishkek__version%202.pdf

[11] Huang Z, Zhao H, Zhang H, Zhang S, Pan T. Social ecological model-based analysis of sport exercisebehaviors and associated factors among children andadolescents in Ningxia. Chin J School Health. 2023;44(02):205–7. 10.16835/j.cnki.1000-9817.2023.02.010.

[12] García MZ, Gil-Madrona P, Prieto-Ayuso A, Garcia DZ. Emociones generadas por distintos tipos de juegos en clase de Educación Física. /International J Med Sci Phys Activity Sport. 2018;18(69):15–40. Revista Internacional de Medicina y Ciencias de la Actividad Física y del Deporte.

[13] Seligman MEP, Csikszentmihalyi M. Positive psychology. An introduction. american psychologist. 2014. 10.1037//0003-066x.55.1.5 11392865

[14] Hamill SK. Resilience and Self-Efficacy: The importance of efficacy beliefs and coping mechanisms in resilient adolescents. 2003.

[15] Mlinac ME, Sheeran TH, Blissmer B, Lees F, Martins D. Psychological Resilience. Med-Leg J. 2016;84(4).

[16] Allred SL, Harrison LD, O’Connell DJ. Self-Efficacy. Prison J. 2013;93:211–33.

[17] Mantz LS, Bear GG, Yang C, Harris A. The delaware social-emotional competency scale (DSECS-S): evidence of validity and reliability. Child Indic Res. 2018;11:137–57.

[18] Shrandt J, Townsend D, Poulson C. Teaching empathy skills to children with autism. J Appl Behav Anal. 2009;42(1):17–32. 19721727 10.1901/jaba.2009.42-17 PMC2649842

[19] Tse AC. Brief report: Impact of a physical exercise intervention on emotion regulation and behavioral functioning in children with autism spectrum disorder. J Autism Dev Disord. 2020;50(11):4191–8. 32130593 10.1007/s10803-020-04418-2

[20] Purnomo E, Jermaina N, Marheni E, Gumilar A, Widarsa AH, Elpatsa A et al. Enhancing problem-solving skills through physical education learning: a comprehensive analysis. Retos: nuevas tendencias en educación física. deporte y recreación. 2024;(58):435–44.

[21] Bulger SM, Townsend JS, Carson LM. Promoting responsible student decision-making in elementary physical education. J Phys Educ Recreation Dance. 2001;72(7):18–23.

[22] Gao Y, Yan J, Chen H, Jiang Y, Lu T, Tao B. Relationship between physical activity and school adjustment in highschool students: The mediating role of interpersonal distress. China J Health Psychol. 2024;32(03):403–9. 10.13342/j.cnki.cjhp.2024.03.015.

[23] Zhong B, Yan J, Tao B, Jiang Y, Lu T, Chen H, et al. The Influence of Moderate Intensity Physical Exercise on InterpersonalConflict Information Perception of College Students Troubled byInterpersonal Relationship. Sports Sci. 2024;45(03):110–20. 10.13598/j.issn1004-4590.2024.03.012.

[24] Caspersen CJ, Powell KE, Christenson GM. Physical activity, exercise, and physical fitness: definitions and distinctions for health-related research. Public Health Rep. 1985;100(2):126. 3920711 PMC1424733

[25] Wang E, Xia M, Qu D, Zhang J, Liang K, Xiao J, et al. The Effect of Prototypical Family Functioning Trajectories on Junio!High School Students’ Internet Addiction: The Mediating Role of Resilience. Psychol Dev Educ. 2024;40(06):853–64. 10.16187/j.cnki.issn1001-4918.2024.06.10.

[26] A N, J P, L J, C S, F C, J CP. The mediating role of mental toughness in sport. J Sports Med Phys Fit. 2015. 26360967

[27] Marshall T, Roberts J, Pack S, Basevitch I, Rossato C, Suckiling C, et al. The effect of long term physical training on the development of mental toughness in recreationally active participants. J Multidisciplinary Res. 2017;9(2):29–43.

[28] Hu Q. The Effect of increased intensity of physical exercises on mental healthand resilience among college students. Chin J School Health. 2019;40(01):83–5. 10.16835/j.cnki.1000-9817.2019.01.022.

[29] Gerber M, Kalak N, Lemola S, Clough PJ, Pühse U, Elliot C, et al. Adolescents’ exercise and physical activity are associated with mental toughness. Ment Health Phys Act. 2012;5(1):35–42.

[30] Styles BW, Williams JJ. Promoting Resilience in Teachers: An Examination of a Program Designed to Improve Teachers’ Social-Emotional Competence. Samford University; 2020.

[31] Aslam S, Saleem S, Mahmood Z, MENTAL TOUGHNESS AND MENTAL HEALTH PROBLEMS IN. DOCTORS: A MEDIATING ROLE OF EMOTION REGULATION. Khyber Med Univ J. 2021;13(1).

[32] Yuan Z, Huang Z, Li J, Zhang J. Report on Chinese Adolescence’s Development of Social and Emotional Skills. J East China Normal University(Educational Sciences). 2021;39(09):1–32. 10.16382/j.cnki.1000-5560.2021.09.001.

[33] Gucciardi DF, Peeling P, Ducker KJ, Dawson B. When the going gets tough: Mental toughness and its relationship with behavioural perseverance. J Sci Med sport. 2016;19(1):81–6. 25554654 10.1016/j.jsams.2014.12.005

[34] Bandura A. Reflections on self-efficacy. Adv Behav Res therapy. 1978;1(4):237–69.

[35] McAuley E, Katula J. Mode of physical activity and self-efficacy in older adults: A latent growth curve analysis. Journals Gerontol. 1999. 10.1093/geronb/54b.5.p283 10542821

[36] Jian J, Gao S, Tang G. The influences of Exercise on The Mental Health of Middle SchoolStudents:Intermediary Effect of Self-efficacy. China Sport Sci Technol. 2016;52(05):98–103. 10.16470/j.csst.201605013.

[37] Bandura A. Self-efficacy: toward a unifying theory of behavioral change. Psychol Rev. 1977;84(2):191. 847061 10.1037//0033-295x.84.2.191

[38] A JI ACP, #X ASBBNO. Connor, Movement competence: Association with physical self-efficacy and physical activity. Hum Mov Sci. 70. 10.1016/j.humov.2020.102582 31957668

[39] Moradi A, Chemelnezhad M. Predicting emotional-social competence based on academic engagement, self-efficacy and perception of school climate in high school students. Iran Evolutionary Educational Psychol J. 2021;3(4):574–82.

[40] Hopf T, Colby N. The relationship between interpersonal communication apprehension and self-efficacy. Communication Res Rep. 1992;9(2):131–5.

[41] Jones SM, Doolittle EJ. Social and emotional learning: Introducing the issue. future Child. 2017;27(1):3–11.

[42] Gecas V. The social psychology of self-efficacy. Ann Rev Sociol. 1989;15(1):291–316.

[43] Gotseva-Balgaranova K. Relations between self-efficacy and social-emotional competence: Evidence from 7-and 8-year-olds in a psychodrama group with children. Z für Psychodrama und Soziometrie. 2023;22(Suppl 2):259–75.

[44] Jiang K, Lan Z, Sun X, Ding X, Tao J. The Influence of Social Support on Academic Self-Efficacy of HearingImpaired College Students:The Mediating Role of PsychologicalResilience. Stud Psychol Behav. 2022;20(01):96–100.

[45] Mary P, Brück A, Li W. Grit Cultivated in Japanese Education: The Foundation of Economic Success and a Comparative Study with the United States. Prim Secondary Schooling Abroad. 1994;(04):1–6.

[46] Schwarzer R, Warner LM. Perceived Self-Efficacy and its Relationship to Resilience. New York: Springer; 2013.

[47] Bingl TY, Batik MV, Hosoglu R, Kodaz AF. Psychological Resilience and Positivity as Predictors of Self-Efficacy. Asian Online J Publishing Group 244 Fifth Avenue Suite D42, New York, NY 10001 Fax: 212-591-6094; e-mail: info@asianonlinejournalscom; Web site: http://wwwasianonlinejournalscom. 2018;(1).

[48] SU P, BS C. A. RQ. The mediating role of self-efficacy in the relationship between resilience and academic performance in adolescence. Learn Motiv. 2022;78.

[49] Liang D. Stress Levels Among College Students and Their Relationship with Physical Exercise. Chin Mental Health J. 1994;(01):5–6.

[50] Pan Y. Home Intergenerational Transmission of Children’s Sports Activities: Mediating Effect of Family Cohesion. J Wuhan Sports Univ. 2022;56(03):38–45. 10.15930/j.cnki.wtxb.2022.03.008.

[51] Huang D, Xiong X. Effects of Physical Activity on Pro-Social Behavior ofRural Left-Behind Children: The LongitudinalMediating Role of Peer Acceptance. J Fujian Normal Univ (Natural Sci Edition). 2025;41(04):116–25.

[52] Zhu X. Study on the reliability and validity of the Chinese version of Delaware Social and Emotional Competency Scale [Master]2016.

[53] Yang J, Deng Y, Wang Y. Reciprocal associations among social–emotional competence, interpersonal relationships and academic achievements in primary school. Behav Sci. 2023;13(11):922. 37998669 10.3390/bs13110922 PMC10669640

[54] Hu Y, Gan Y. Development and Psychometric Validity of the Resilience Scale forChinese Adolescents. Acta Physiol Sinica. 2008;(08):902–12.

[55] Tan L, Sun K, Chu W. Study on the influencing factors of psychological resilience of rural left-behind children. Chin J Health Educ. 2023;39(09):858–63. 10.16168/j.cnki.issn.1002-9982.2023.09.015.

[56] Huang W, Wu P, Sun Y. Universal mental intervention for children in school based on mental resilience-forced enhancement:a randomized controlled trial studyprotocol. Chin J School Health. 2023;44(07):969–73. 10.16835/j.cnki.1000-9817.2023.07.003.

[57] Wang C, Hu Z, Liu Y. Evidences for Reliability and Validity of the Chinese Version of General Self Efficacy Scale. Chin J Appl Psychol. 2001;(01):37–40.

[58] Li X, Li Z, Li X. Mediating effect of family intimacy on resilience in left-behind children. China J Health Psychol. 2021;29(03):387–91. 10.13342/j.cnki.cjhp.2021.03.014.

[59] Yuan Y, Wu M, Wang Z, Li Z. Family Socioeconomic Status and Children’s General Self-efficacy: ChainMediation of Parents’ Care and Coping Style. Chin J Clin Psychol. 2020;28(05):1009–12. 10.16128/j.cnki.1005-3611.2020.05.030.

[60] Subarkah A, editor. Analysis of interpersonal communication in sports. 2nd Yogyakarta International Seminar on Health, Physical Education, and Sport Science (YISHPESS 2018) and 1st Conference on Interdisciplinary Approach in Sports (CoIS 2018); 2018: Atlantis Press.

[61] Wu J, Zhu L, Dong X, Sun Z, Cai K, Shi Y, et al. Relationship between physical activity and emotional regulation strategies in early adulthood: mediating effects of cortical thickness. Brain Sci. 2022;12(9):1210. 36138946 10.3390/brainsci12091210 PMC9496840

[62] Zimmermann L. The influence of physical activity on information: processing in consumer decision making. London School of Economics and Political Science; 2017.

[63] Moreira M, Veiga G, Lopes F, Hales D, Luz C, Cordovil R. Kindergarten affordances for physical activity and preschoolers’ motor and social-emotional competence. Children. 2023;10(2):214. 36832343 10.3390/children10020214 PMC9955055

[64] Madrona PG, Pascual-Frances L, Jordá-Espi A, Johnson FNM, Revelles ABF. Affectivity and Motor Interaction in Popular Motor Games at School. Apunts Educ Fisica y Deportes. 2020;139:42–8.

[65] Li J, Shao W. Influence of sports activities on prosocial behavior of children and adolescents: A systematic literature review. Int J Environ Res Public Health. 2022;19(11):6484. 35682069 10.3390/ijerph19116484 PMC9180162

[66] Gill EL. Mental health in college athletics: It’s time for social work to get in the game. Soc Work. 2008;53(1):85–8. 18610824 10.1093/sw/53.1.85

[67] Ozbay F, Johnson DC, Dimoulas E, Morgan Iii C, Charney D, Southwick S. Social support and resilience to stress: from neurobiology to clinical practice. Psychiatry (edgmont). 2007;4(5):35. 20806028 PMC2921311

[68] Eskandarnejad M. Physical Activity and Mental Toughness in University Students of Tabriz University. Res J Sport. 2015;3(8):226–32.

[69] Belcher BR, Zink J, Azad A, Campbell CE, Herting MM. The roles of physical activity, exercise, and fitness in promoting resilience during adolescence: effects on mental well-being and brain development. Biological Psychiatry: Cognitive Neuroscience and Neuroimaging; 2020. 10.1016/j.bpsc.2020.08.005 PMC7878276 33067166

[70] Maddi SR, Hardiness. The courage to grow from stresses. J Posit Psychol. 2006;1(3):160–8.

[71] Zhang Z. The Relationship Between Learning Burnout, Personality Hardiness, and Physical Exercise. China Adult Educ. 2011;(08):131–3.

[72] Xi J, Zuo Z, Wei W. Daily emotional states and emotional self-regulation strategies amonghigh school students with different resilience levels. Chin Mental Health J. 2013;27(09):709–14.

[73] Çiftçi İ. The effect of psychological resilience and social/emotional competence on communication skills. Synesis (ISSN 1984–6754). 2023;15(4):331–44.

[74] Tang Z. On the Relationship Between Physical Exercise and Mental Health. J Psychol Sci. 2000;03370–69. 10.16719/j.cnki.1671-6981.2000.03.033.

[75] Bandura A, Freeman WH, Lightsey R. Self-Efficacy: The Exercise of Control. J Cogn Psychother. 1997.

[76] Tang D, Dong Y, Yu G, Wen S. The Regulatory Emotional Self-Efficacy:A New Research Topic. Adv Psychol Sci. 2010;18(04):598–604.

[77] Zhang D. A Survey of General Self-Efficacy and Interpersonal Skills Among Vocational College Students. Chin Vocat Tech Educ. 2009;(10):60–1.

[78] Langermann A. Togetherness and the Belief in Oneself: Investigating Self-Efficacy within the Context of Community Resilience. University of Twente; 2023.

[79] Peng B, Chen W, Wang H, Yu T. How does physical exercise influence self-efficacy in adolescents? A study based on the mediating role of psychological resilience. BMC Psychol. 2025;13(1):1–17. 40119462 10.1186/s40359-025-02529-y PMC11927186

[80] Navickienė O, Vasiliauskas AV. The effect of cadet resilience on self-efficacy and professional achievement: verification of the moderated mediating effect of vocational calling. Front Psychol. 2024;14:1330969. 38259580 10.3389/fpsyg.2023.1330969 PMC10800948

